# Pricing Analysis of Online Shopping Platforms Considering Consumer Information Levels

**DOI:** 10.3389/fpsyg.2022.821979

**Published:** 2022-03-21

**Authors:** Hao Chen, Weiqing Xiong, Peichen Xiong

**Affiliations:** ^1^Business Faculty of Ningbo University, Ningbo, China; ^2^Zhejiang University of Finance and Economics Dongfang College, Haining, China

**Keywords:** two-sided platform, information asymmetry, consumer information, cross-network externalities, return measures

## Abstract

To address the problem of frequent dishonest transactions by online shopping platform merchants, we developed monopoly and competitive platform pricing models based on two-sided market theory, which introduce consumer information levels. This article analyzes the incentives of the platforms to improve consumer information levels in platform pricing strategies. Monopoly online shopping platforms aim to maximize profits. The higher the consumer information level is, the lower the fees charged to merchants; this can lead to increased platform profits. The charging of consumers depends on cross-network externalities. Competitive online shopping platforms also aim at maximizing profits. Under the circumstance that the number of consumers remains the same, the higher the consumer information level is, the more merchants the platforms will attract. This reduces bilateral user fees, and platform profits will be lower. From the perspective of consumer information level, the article analyzes the impact of monopoly and competitive platforms adopting return measures to improve the level of consumer information on platform pricing, number of bilateral users, and profits.

## Introduction

The evolution of business involves overcoming information asymmetries and credit non-delivery. In the process, it reduces transaction costs, increases network density, and enhances transaction efficiency. Various online shopping platforms, such as eBay, Amazon, Taobao, JD, and Guazi, act as connecting intermediaries, enabling consumers and merchants to enact transactions across time and space, thereby enhancing convenience. Merchants have natural private information about goods (Daughety and Reinganum, 2008), resulting in information asymmetries for bilateral users, leading to dishonesty in online shopping platforms (such as for commodity quality), which is why consumers choose to leave the platform company. With cross-network externalities, the number of merchants is affected, and so is the pricing strategy of online shopping platforms (Rochet and Tirole, 2003; Armstrong and Wright, 2006; Feng et al., 2020).

In response to the dishonest behavior of merchants, the platform adopts prior mechanisms, such as merchant certifications, ratings, and interactive consumer reviews, to make merchants disclose more product information and improve consumer product information levels, which reduces bilateral user information asymmetries. To further improve the online transaction ex-post mechanism, the platforms have introduced diversified return services to enhance consumer information level. For example, employing 7 days of no-reason return service, the Tmall trading platform has upgraded its return service for consumers who encounter problems with the quality of goods. It has successively launched a 360-warranty service, a 30-day no-worry-return service, and a 30-day warranty plus service. JD launched a 1-year warranty service and a 3-year warranty service to protect consumer rights.

Given the above background, as well as Armstrong's (2006) complete information pricing model and Hagiu and Hałaburda's (2014) incomplete information pricing model that considers the degree of user expectations, this study introduces the level of consumer information to build a platform pricing model with incomplete information. The primary goal is to analyze the effect of consumer information levels on online shopping platform pricing strategies under different market structures. We explain why platforms adopt measures (such as returns) to increase the level of consumer information and reduce the level of bilateral information asymmetry. The following questions are addressed: (1) what is the impact of consumer information level on pricing strategies of online shopping platforms with different market structures under the effect of cross-network externalities, and how does it increase profits?, and (2) what is the impact on pricing strategies when platforms adopt information level improvement services (such as return services)?

By platform profit maximization, we analyze the effect of consumer information level on platform pricing for bilateral users, number of bilateral users, and platform profit. We find that increasing the level of consumer information in the competitive platform reduces platform profits, resulting in less incentive for the competitive platform to increase the level of consumer information. In contrast, the monopoly platform has a greater incentive to increase the level of consumer information. Platforms set different return times for different product categories to improve consumer information levels and ensure consumer rights.

Other parts of this study are as follows. section Literature Review reviews the relevant literature. section Model Assumptions explains model construction. Section Model Analysis and Results analyzes the impact of consumer information level on optimal pricing, bilateral user size, and platform profit in different market structures. Section Discussion further discusses changes in the platform's pricing strategy when the platform adopts return measures to improve its information service level. Section Conclusion concludes the study with main conclusions and some recommendations for future research.

## Literature Review

Our study is mainly related to three streams of existing research: two-sided market pricing, user expectation management, and platform governance mechanisms.

Bilateral platform pricing is largely based on the models of Rochet and Tirole (2003) and Armstrong (2006). There is one pricing model type for monopoly and competitive platforms from the perspective of the relationship between price and demand (Rochet and Tirole, 2002, 2003). A second type builds a pricing model of monopoly platforms and competitive platforms from the perspective of cross-network externalities (Armstrong, 2006). In early studies, platforms struggled to create cross-network effects because of high cost of consumers joining online platforms, leading to chicken-and-egg problems (Rohlfs, 1974; Caillaud and Jullien, 2003). There are two ways to address this. The first approach is that a platform uses price subsidies to increase independent values when consumers join a platform (Amelio and Jullien, 2012). With small marginal costs and high elasticity of demand on one side of the digital platform, to enable that side to have higher user participation, the platform implements price subsidies or free policies for users (Zhao et al., 2021). The second approach is that platforms adopt a vertical integration strategy to increase bilateral user interaction benefits (Hagiu and Spulber, 2013). For example, Google has introduced application development software for the Android platform (Wen and Zhu, 2019). As competition between platforms becomes more intense, bilateral platforms (such as those for dating, e-commerce, logistics, and media) emerge to meet the diverse needs of consumers. Platforms provide differentiated services or products for consumers, at which time bilateral users can either choose to join the platform with single- or multi-homing.

Bilateral market research under information asymmetry involves user expectation management. Maskin and Riley (1984) analyzed monopoly and competitive markets with incomplete information. In a study on the telecommunication market with network externalities, and the passive expectation concept of the unilateral network effect was proposed considering the rational expectation of consumers (Katz and Shapiro, 1985, 1986). Based on Katz and Shapiro's assumption of rational expectations, as followers enter, the position of the network leader is undermined, and market competition becomes more intense (Economides, 1996). In highly competitive markets, competitive platforms do not disclose advanced information to consumers and competitors from the perspective of profit and welfare maximization, although they increase expected network effects through advanced information strategies (Chellappa and Mukherjee, 2021). Only monopolistic platforms take advantage of information to improve pricing with the cross-network effect, and the platform pricing of passive expectation is lower than that of responsive expectation (Hagiu and Hałaburda, 2014; Hurkens and López, 2014). Such studies have studied platform pricing strategies with information asymmetry, but they did not consider which mechanisms could reduce information asymmetry and, thus, change platform pricing strategies.

Information asymmetry raises moral hazard and adverse selection problems (Akerlof, 1970; Stiglitz, 1983). To reduce information asymmetry, platform companies often adopt information disclosure, such as reputation mechanisms, margin mechanisms, and signaling. Airbnb improves consumer trust and facilitates bilateral user transactions through information disclosure methods, such as merchant response rate, merchant verification information, and overall consumer score (Xu et al., 2021). Online shopping platforms invest in developing information disclosure tools (Zhang et al., 2018) and establishing reputation feedback systems (Tadelis, 2016) to facilitate consumer purchasing decisions. Crowdfunding platforms use media information and crowdfunding experience to reduce the degree of information asymmetry between sponsors and funders to achieve project funding objectives (Courtney et al., 2016). In addition, E-commerce platforms also provide different deposit policies to restrain dishonest transactions and improve trust in the platforms (Wang L. et al., 2021). Based on the transmission theory of labor markets (Spence, 1973), a platform can use advertising, full returns and prices to convey signals of high-quality products and reduce information asymmetry before and after transactions (Kihlstrom and Riordan, 1984; Moorthy and Srinivasan, 1995). In addition to the common mechanisms mentioned above, government regulation (Han, 2018) and technological progress of platform enterprises (Babich and Hilary, 2020; Wang Y. et al., 2021) can reduce information asymmetry. Although platform firms play a crucial role in reducing bilateral user information asymmetry by adopting these governance mechanisms, they do not consider platform network effects.

In sum, existing studies mainly focus on platform pricing with complete information and less research on platform pricing with incomplete information. In both response expectation and passive expectation scenarios, Hagiu and Hałaburda (2014) focus on the impact of user expectation changes on market equilibrium pricing and platform profits. In contrast to the study by Hagiu and Hałaburda (2014), we study the impact of consumer information level on platform pricing, number of bilateral users, and platform profits for monopoly online shopping platforms, competitive online shopping platforms, and incomplete competition online shopping platforms. Moreover, we further analyze pricing changes induced when platforms decrease information asymmetry of bilateral users (e.g., by adopting return measures).

## Model Assumptions

In this section, following Armstrong (2006) and Hagiu and Hałaburda (2014), we introduce consumer information levels and build monopoly and competitive platform pricing models. The following assumptions are made, and the notation needed for modeling is defined in Table 1.

**Table 1:** Definition of notations.

**Notations**	**Definition**
*v*	Merchant-to-consumer network externalities (*v* ∈ [0, 1])
φ	Consumer-to-merchant network externalities (φ ∈ [0, 1])
*V*_0_	The utility of the base services provided by the platform to consumers
θ	Consumer information level (θ ∈ [0, 1])
*c*	The cost of goods or services provided by the merchant to the consumer
*p*_*b*_, *p*_*s*_	Membership fees charged to consumers and merchants in the monopoly platform
*p*_*ib*_, *p*_*is*_	Membership fees charged to consumers and merchants in the competitive platform *i*(*i* = 1, 2)
*N*_*b*_, *N*_*s*_	Number of consumers and merchants in the monopoly platform
*N*_*ib*_, *N*_*is*_	Number of consumers and merchants in the competitive platform *i*(*i* = 1, 2)
$N_{s}^{e},N_{is}^{e}$	The number of merchants expected by consumers in the monopoly platform and competitive platform i(i = 1,2)
Π_*pl*_Π_*pli*_	Monopoly platform profit, competitive platform *i*(*i* = 1, 2) profit

Assumption 1. Extending the network externality theory of two-sided markets, several existing studies are used to simplify the model (Armstrong, 2006; Armstrong and Wright, 2006; Dou et al., 2020). Therefore, we assume that the scale of consumers and merchants is normalized to 1 and that there are cross-network externalities of platform bilateral users without considering intragroup network externalities. Each merchant generates utility for each consumer *v*(*v* ∈ [0, 1]), and each consumer generates utility for each merchant φ(φ ∈ [0, 1] ).

Assumption 2. According to the information asymmetry theory, which is different from the theory on which the Armstrong (2006) model is based, we consider the heterogeneity of consumers in online trading platforms. To this end, we design two groups of consumers, informed and uninformed, to study platform pricing strategies (Chao and Derdenger, 2013; Hagiu and Hałaburda, 2014; Dou et al., 2020). When bilateral users are fully informed, the number of merchants expected by consumers is equal to the number of actual merchants, i.e., $N_{s}^{e}=N_{s}$; the number of consumers expected by merchants is equal to the number of actual consumers, i.e., ${N_{b}^{e}=N}_{b}$. The reason for this assumption is that, in reality, consumers, and merchants have asymmetric pricing information for each other. A merchant usually knows the pricing information of the platform with respect to consumers and the consumers' needs, i.e., ${N_{b}^{e}=N}_{b}$. However, consumers do not understand pricing information such as transaction fees and advertising fees, charged by a platform. Consumers can only expect merchants to enter based on their reputation, sales volume, and other information; therefore, when consumers have complete information about merchants, $N_{s}^{e}=N_{s}$.

In the monopoly platform, the consumer utility function consists of payment of membership fees *p*_*b*_ by consumers and consumers getting the base service utility *V*_0_ (e.g., the platform provides product browsing and accurate search results), and the merchant provides the informed consumer utility *vN*_*s*_ (uninformed consumer utility$vN_{s}^{e}$), as shown in Equation (1):


(1) $$\begin{array}{l} U_{b\left(Informed\right)}=V_{0}+vN_{s}-p_{b},U_{b\left(Uninformed\right)\;}=V_{0}+vN_{s}^{e}-p_{b} \end{array}$$

When consumer utility *U*_*b*(*i*)_ (*i* = *Informed, Uninformed*) ≥ 0, i.e., α ≥ *p*_*b*_ − *vN*_*s*_, $\alpha \geq p_{b}-vN_{s}^{e}$, consumers choose to enter a platform transaction. Then, the number of informed and uninformed consumers entering the platform transaction is:


(2) $$\begin{array}{l} N_{b\left(Informed\right)}=1+vN_{s}-p_{b},N_{b\left(Uninformed\right)}=1+vN_{s}^{e}-p_{b} \end{array}$$

Because of information asymmetry between consumers and merchants, the consumer quantity function consists of two components, which are the θ proportion of informed consumers and the 1 − θ proportion of uninformed consumers, as shown in Equation (3):


(3) $$\begin{array}{l} N_{b}=\theta \left(1+vN_{s}-p_{b}\right)+\left(1-\theta \right)\left(1+vN_{s}^{e}-p_{b}\;\right) \end{array}$$

The higher the information service level provided by the platform, the more information (on product quality, sales volume, reputation, etc.) merchants can deliver to consumers; that is, the higher the consumer information level θ, the higher the proportion of informed consumers [ *N*_*b*(*Informed*)_] and the lower the proportion of uninformed consumers [ *N*_*b*(*Uninformed*)_].

Similarly, the merchant utility function consists of the membership fee *p*_*s*_ paid by merchants, the cost *c* of providing a product or service to consumers, and the utility φ*N*_*b*_ brought by consumers to merchants, as shown in Equation (4):


(4) $$\begin{array}{l} U_{s}=\varphi N_{b}-p_{s}-c \end{array}$$

A merchant chooses to enter a platform transaction only when merchant utility *U*_*s*_ ≥ 0, i.e., φ*N*_*b*_ − *p*_*s*_ ≥ *c*. Then, the function of the number of merchants entering platform transactions is calculated by Equation (5):


(5) $$\begin{array}{l} N_{s}=\varphi N_{b}-p_{s} \end{array}$$

The monopoly platform profit function is shown in Equation (6):


(6) $$\begin{array}{l} \Pi_{pl}=p_{b}N_{b}+p_{s}N_{s} \end{array}$$

In the competitive platform, we use the Hotelling model to describe the competition between symmetric platforms 1 and 2. The two platforms are located at the two ends of a line segment [0, 1], and bilateral users are distributed uniformly on the line segment. Considering that the unit search cost of bilateral users is not the focus of this model, the simplified unit search cost is 1.

In the competitive platform, because bilateral user information asymmetry, the consumer quantity function still consists of two components, which are the θ proportion of informed consumers and 1 − θ proportion of uninformed consumers, as shown in Equations (7) and (8):


(7) $$\begin{array}{l} N_{1b}=\theta \left(\frac{1}{2}+\frac{v\left(N_{1s}-N_{2s}\right)-\left(p_{1b}-p_{2b}\right)}{2}\right)\\ \;+\left(1-\theta \right)\left(\frac{1}{2}+\frac{v\left(N_{1s}^{e}-N_{2s}^{e}\right)-\left(p_{1b}-p_{2b}\right)}{2}\;\right) \end{array}$$

(8) $$\begin{array}{l} N_{2b}=1-N_{1b} \end{array}$$

Here, $\frac{1}{2}+\frac{v\left(N_{1s}-N_{2s}\right)-\left(p_{1b}-p_{2b}\right)}{2}$ denotes the number of informed consumers in the competitive platform, and $\frac{1}{2}+\frac{v\left(N_{1s}^{e}-N_{2s}^{e}\right)-\left(p_{1b}-p_{2b}\right)}{2}$ denotes the number of uninformed consumers in the competitive platform.

In the competitive platform, the merchants' number function consists of bilateral cross-network utilities and membership fees charged by the platform to merchants, as shown in Equations (9) and (10):


(9) $$\begin{array}{l} N_{1s}=\varphi N_{1b}-p_{1s} \end{array}$$

(10) $$\begin{array}{l} N_{2s}=\varphi N_{2b}-p_{2s} \end{array}$$

Then, the profit function of the competitive platform *i* is calculated with Equation (11):


(11) $$\begin{array}{l} \Pi_{pli}=p_{ib}N_{ib}+p_{is}N_{is}\left(i=\;1,2\right). \end{array}$$

## Model Analysis and Results

### Analyzing the Effect of Consumer Information Level in the Monopoly Platform

**Result 1** The higher the consumer information level, the more the monopoly platform profits under the condition of cross-network externalities.

**Proof** Substituting Equation (5) into Equation (3) gives Equation (12):


(12) $$\begin{array}{l} N_{b}=\frac{1+\left(1-\theta \right)\;vN_{s}^{e}-\theta vp_{s}-p_{b}}{1-\theta \varphi v},\\ N_{s}=\frac{\varphi +\left(1-\theta \right)\;\varphi vN_{s}^{e}-p_{s}-\varphi p_{b}}{1-\theta \varphi \;v} \end{array}$$

Substituting Equation (12) into Equation (6), first-order derivatives are taken for *p*_*b*_ and *p*_*s*_, as shown in Equation (13):


(13) $$\begin{array}{l} \frac{\partial \Pi }{\partial p_{b}}=\frac{1+\left(1-\theta \right)\;vN_{s}^{e}-\left(\varphi +\theta v\right)\;p_{s}-{2p}_{b}}{1-\theta \varphi v},\\ \frac{\partial \Pi }{\partial p_{s}}=\frac{\varphi +\left(1-\theta \right)\;vN_{s}^{e}-\left(\varphi +\theta v\right)\;p_{b}-{2p}_{s}}{1-\theta \varphi \;v}. \end{array}$$

Let $\frac{\partial \Pi }{\partial p_{b}}=0$ and $\frac{\partial \Pi }{\partial p_{s}}=0$. Under the$N_{s}=N_{s}^{e}$ equilibrium condition, the platform's optimal pricing for consumers and the platform's optimal pricing for merchants are given in Equation (14):


(14) $$\begin{array}{l} p_{b}^{*}=\frac{2-\varphi \;(\varphi +\theta v)\;}{4-\;(\varphi +v)\;\;(\varphi +\theta v)\;},p_{s}^{*}=\frac{\varphi -\theta v}{4-\;(\varphi +v)\;\;(\varphi +\theta v\;)\;}. \end{array}$$

Substituting Equation (14) into Equation (12), the platform's optimal number of bilateral users is given in Equation (15):


(15) $$\begin{array}{l} N_{b}^{*}=\frac{2}{4-\;(\varphi +v)\;\;(\varphi +\theta v)\;},N_{s}^{*}=\frac{\varphi +\theta v}{4-\;(\varphi +v)\;\;(\varphi +\theta v\;)\;} \end{array}$$

Substituting Equation (14) and Equation (15) into Equation (6), the monopoly platform's optimal profit is given in Equation (16):


(16) $$\begin{array}{l} \Pi_{pl}^{*}=\frac{4-{\;(\varphi +\theta v)\;}^{2}}{{\left[4-\;(\varphi +v)\;\;(\varphi +\theta v)\;\;\right]}^{2}} \end{array}$$

The monopoly platform's optimal profit takes the first-order derivative for consumer information level, as shown in Equation (17):


(17) $$\begin{array}{l} \frac{\partial \Pi_{pl}^{*}}{\partial \theta }=\frac{8v^{2}\;(1-\theta )\;\left[4-\;(\varphi +v)\;\;(\varphi +\theta v)\;\right]}{{\left[4-\;(\varphi +v)\;\;(\varphi +\theta v)\;\right]}^{4}}>\;0 \end{array}$$

Equation (17) shows that the higher the consumer information level, the higher the monopoly platform profit. From Equation (16), when θ = 0, there is highest level of asymmetry between consumers and merchants and the lowest proportion of informed consumers, the lowest profit of the monopoly platform is $\frac{4-\varphi^{2}}{{\left[4-\varphi \left(\varphi +v\right)\right]}^{2}}$; when θ = 1, there is lowest level of asymmetry between consumers and merchants, and the highest proportion of informed consumers, and the highest profit of the monopoly platform is $\frac{4-{\left(\varphi +v\right)}^{2}}{{\left[4-{\left(\varphi +v\right)}^{2}\right]}^{2}}$.

It follows from result 1 that for the monopoly online shopping platform, the higher consumer information level, i.e., the lower bilateral user information asymmetry, the higher the online shopping platform profit.

**Result 2** In the monopoly platform, the effect of consumer information level on platform-to-consumer pricing is decided by the magnitude of cross-network externalities; the higher the consumer information level, the lower the platform-to-merchant pricing.

**Proof** Bilateral user pricing in monopoly platforms takes the first-order derivative for consumer information level, as shown in Equation (18):


(18) $$\begin{array}{l} \frac{\partial p_{b}^{*}}{\partial \theta }=\frac{2v\;(v-\varphi )\;}{{\left[4-\;(\varphi +v)\;\;(\varphi +\theta v)\;\right]}^{2}},\\ \frac{\partial p_{s}^{*}}{\partial \theta }=\frac{\left[2\varphi \;(\varphi +v)\;-4\right]v}{{\left[4-\;(\varphi +v)\;\;(\varphi +\theta v)\;\right]}^{2}}<\;0 \end{array}$$

Equation (18) shows that if merchant network externality is greater than consumer network externality, the higher the consumer information level and the higher the monopoly platform's pricing to consumers; if merchant network externality is less than consumer network externality, the higher the consumer information level, the lower the monopoly platform's pricing to consumers. When consumer information level increases, the monopoly platform pricing to merchants decreases.

**Result 3** The higher the consumer information level, the more the platform attracts bilateral users to join under cross-network externalities of the monopoly platform.

**Proof** In the monopoly platform, the number of bilateral users takes the first-order derivative for consumer information level, as shown in Equation (19):


(19) $$\begin{array}{l} \frac{\partial N_{b}^{*}}{\partial \theta }=\frac{2v\;(v+\varphi )\;}{{\left[4-\;(\varphi +v)\;\;(\varphi +\theta v)\;\right]}^{2}}>0,\\ \frac{\partial N_{s}^{*}}{\partial \theta }=\frac{4v}{{\left[4-\;(\varphi +v)\;\;(\varphi +\theta v)\;\right]}^{2}}>\;0 \end{array}$$

With cross-network externalities, Equation (19) shows that as consumer information level increases, the number of monopoly platform bilateral users increases, and that consumers and merchants are more willing to transact on the platform.

According to result 2 and result 3, Equation (18) and Equation (19) are subtracted, as shown in Equation (20).


(20) $$\begin{array}{l} \frac{\partial p_{b}^{*}}{\partial \theta }-\frac{\partial N_{b}^{*}}{\partial \theta }<0,\frac{\partial p_{s}^{*}}{\partial \theta }-\frac{\partial N_{s}^{*}}{\partial \theta }<\;0 \end{array}$$

From Equation (20), the effect of consumer information level on the number of monopoly platform bilateral users is greater than the effect on bilateral user pricing. Therefore, regardless of cross-network externality magnitude, increasing consumer information levels will eventually lead to increase in platform profits.

In sum, the monopoly platform aims at maximizing profit given cross-network externalities. It increases consumer information levels and brings increased benefits to consumers, merchants, and the platform, which improves overall social welfare. Therefore, the monopoly platform has an incentive to increase consumer information level.

### Analyzing the Effect of Consumer Information Level in the Competitive Platform

**Result 4** With cross-network externalities of the competitive platform, the lower the consumer information level, the higher the platform profits.

**Proof** Substituting Equation (8), Equation (9), and Equation (10) into Equation (7) yields Equation (21).


(21) $$\begin{array}{l} N_{1b}=\frac{1-\theta v\;(\varphi +p_{1s}-p_{2s})\;+\;(1-\theta )\;\;v\;(N_{1s}^{e}-N_{2s}^{e})\;+p_{2b}-p_{1b}}{2\;(1-\theta \varphi v\;)\;} \end{array}$$

Substituting Equation (9) and Equation (21) into Equation (11) yields Equation (22):


(22) $$\begin{array}{l} \Pi_{pl1}=\;(p_{1b}+\varphi p_{1s})\;\\ \frac{1-\theta v\;(\varphi +p_{1s}-p_{2s})\;+\;(1-\theta )\;\;v\;(N_{1s}^{e}-N_{2s}^{e})\;+p_{2b}-p_{1b}}{2\;(1-\theta \varphi v)\;}\\ -{p_{1s}}^{2} \end{array}$$

In Equation (22), platform profit takes the first-order derivative separately for bilateral user pricing, as shown in Equation (23):


(23) $$\begin{array}{l} \frac{\partial \Pi_{pl1}}{\partial p_{1b}}=\frac{1-\theta v\;(\varphi +p_{1s}-p_{2s})\;+\;(1-\theta )\;\;v\;(N_{1s}^{e}-N_{2s}^{e})\;+p_{2b}-2p_{1b}-\varphi p_{1s}}{2\;(1-\theta \varphi v\;)\;},\\ \frac{\partial \Pi_{pl1}}{\partial p_{1s}}=\frac{\varphi \;(1-\theta v)\;\;(\varphi +p_{1s}-p_{2s})\;+\;(1-\theta )\;\;\varphi v\;(N_{1s}^{e}-N_{2s}^{e})\;+\varphi \;(p_{2b}-p_{1b})\;-\theta v\;(p_{1b}+\varphi p_{1s})\;}{2\;(1-\theta \varphi v)\;}-\;2p_{1s} \end{array}$$

Let $\frac{\partial \Pi_{pl1}}{\partial p_{1b}}=0$ and $\frac{\partial \Pi_{pl1}}{\partial p_{1s}}=0$. According to the Armstrong (2006) model, because of the symmetry of the platform, platform pricing for bilateral users satisfies *p*_1*b*_ = *p*_2*b*_ and *p*_1*s*_ = *p*_2*s*_ under the $N_{is}=N_{is}^{e}$ equilibrium condition, and we obtain competitive platform optimal pricing for consumers and optimal pricing for merchants, as shown in Equation (24):


(24) $$\begin{array}{l} p_{b}^{*}=\frac{4-3\theta \varphi v-\varphi^{2}}{4},p_{s}^{*}=\frac{\varphi -\theta v}{4}. \end{array}$$

Substituting Equation (24) into Equation (7), Equation (8), Equation (9), and Equation (10) gives the optimal number of consumers and merchants for the competitive platform, as shown in Equation (25):


(25) $$\begin{array}{l} N_{b}^{*}=\frac{1}{2},N_{s}^{*}=\frac{\varphi +\;\theta v}{4}. \end{array}$$

Substituting Equation (24) and Equation (25) into Equation (11) gives the optimal profit of the competitive platform, as shown in Equation (26):


(26) $$\begin{array}{l} \Pi_{pl}^{*}=\frac{8-\varphi^{2}-6\theta \varphi v-\;\theta^{2}v^{2}}{16}. \end{array}$$

The competitive platform profit takes the first-order derivative for consumer information level, as shown in Equation (27):


(27) $$\begin{array}{l} \frac{\partial \Pi_{pl}^{*}}{\partial \theta }=\frac{-6\varphi v-2\theta v^{2}}{16}<\;0 \end{array}$$

From Equation (27), under the effect of cross-network externalities, the higher the consumer information level, the lower the competitive platform profit. From Equation (26), when θ = 0 with the highest level of asymmetry between consumers and merchants and the lowest proportion of single-homing informed consumers, then the highest competitive platform profit is $\frac{8-\varphi^{2}}{16}$; when θ = 1 with lowest level of asymmetry between consumers and merchants and highest proportion of single-homing informed consumers, then the lowest competitive platform profit is $\frac{8-\varphi^{2}-6\varphi v-v^{2}}{16}$.

From result 4, the lower the consumer information level, the higher the competitive online shopping platform profit.

**Result 5** With cross-network externalities of the competitive platform, the lower the consumer information level, the higher the platform pricing for bilateral users.

**Proof** Competitive platform pricing for bilateral users takes the first-order derivative for consumer information level, as shown in Equation (28):


(28) $$\begin{array}{l} \frac{\partial p_{b}^{*}}{\partial \theta }=\frac{-3\varphi v}{4}<0,\frac{\partial p_{s}^{*}}{\partial \theta }=\frac{-v}{4}<\;0 \end{array}$$

**Result 6** With cross-network externalities of the competitive platform, consumer information level does not affect the number of consumers; the higher the consumer information level, the more merchants that want to join.

**Proof** The number of consumers and number of merchants in the competitive platform take the first-order derivative for consumer information level, as shown in Equation (29).


(29) $$\begin{array}{l} \frac{\partial N_{b}^{*}}{\partial \theta }=0,\frac{\partial N_{s}^{*}}{\partial \theta }=\frac{v}{4}>\;0 \end{array}$$

The general intuition is that two online shopping platforms compete to attract bilateral users to join the platform by reducing the information asymmetry of the bilateral users. The platform decreases pricing for bilateral users, which makes the competing platforms more profitable. However, this conclusion is the opposite of result 4. Based on calculations in Equation (28) and Equation (29), the competitive platform exploits the information asymmetry of bilateral users to increase profits. The competitive platform maximizes profits by increasing the information asymmetry of bilateral users and decreasing pricing to bilateral users.

In the above analysis, the competitive platform aims to maximize profits by reducing consumer information level and increasing the fees charged to bilateral users under cross-network externalities. Therefore, the competitive platform has no incentive to raise consumer information level.

### Analyzing the Effect of Consumer Information Level in the Mixed Market Structure

Online trading platforms include both head market platforms (e.g., Taobao and JD) and many segmented market platforms (e.g., Mogu and Beibei). Therefore, there is a hybrid state of imperfectly competitive markets between duopoly market structure and monopoly market structure, and this hybrid market structure is dynamic. The consumer quantity function in the hybrid market structure is shown in Equation (30) and Equation (31). The merchant quantity function is given in Equation (9) and Equation (10); the profit function is still calculated using Equation (11):


(30) $$\begin{array}{l} N_{1b}=\theta [\gamma \;(\frac{1}{2}+\frac{v\;(N_{1s}-N_{2s})\;-\;(p_{1b}-p_{2b})\;}{2})\;\\ \;+\;(1-\gamma )\;\;(1+vN_{1s}-p_{1b})\;]+\;(1-\theta )\;\\ \;[\gamma \;(\frac{1}{2}+\frac{v\;(N_{1s}^{e}-N_{2s}^{e})\;-\;(p_{1b}-p_{2b})\;}{2})\;+\;(1-\gamma )\;\\ \;(1+vN_{1s}^{e}-p_{1b})\;\;] \end{array}$$

(31) $$\begin{array}{l} N_{2b}=\theta [\gamma \;(\frac{1}{2}-\frac{v\;(N_{1s}-N_{2s})\;-\;(p_{1b}-p_{2b})\;}{2})\;\\ \;+\;(1-\gamma )\;\;(1+vN_{2s}-p_{2b})\;]+\;(1-\theta )\;\\ \;[\gamma \;(\frac{1}{2}-\frac{v\;(N_{1s}^{e}-N_{2s}^{e})\;-\;(p_{1b}-p_{2b})\;}{2})\;+\;(1-\gamma )\;\\ \;(1+vN_{2s}^{e}-p_{2b})\;\;] \end{array}$$

Here, γ denotes market competition level, and different values of γ indicate market structures at varying times. When γ = 0, the platform has a monopoly market structure, as in case 4.1; when γ = 1, the platform has a duopoly market structure, as in case 4.2.

**Proof** Similar to the proof of 4.2, the solution of the joint cubic Equation (9), Equation (10), Equation (30), and Equation (31) can be obtained as*N*_1*b*_(θ, γ), *N*_2*b*_(θ, γ), *N*_2*s*_(θ, γ), and *N*_2*s*_(θ, γ). Next, we substitute *N*_1*b*_(θ, γ), *N*_2*b*_(θ, γ), *N*_1*s*_(θ, γ), and *N*_2*s*_(θ, γ) into the profit function and take the first-order derivative for the profit function. Let $N_{1s}^{e}=N_{2s}^{e}=N_{s}^{e}$. We can obtain $p_{b}^{*}$, $p_{s}^{*}$, $N_{b}^{*}$, $N_{s}^{*}$, and $\Pi_{pl}^{*}$, as shown in Equation (32):


(32) $$\begin{array}{l} p_{b}^{*}=\frac{BC-\varphi AC}{H};p_{s}^{*}=\frac{AC}{H};N_{b}^{*}=\frac{ABC}{H};N_{s}^{*}=\frac{\varphi ABC-AC}{H};\\ \;\Pi_{pl}^{*}=\frac{AB^{2}C^{2}-A^{2}C^{2}}{H^{2}} \end{array}$$

Here, $A=\frac{2-\gamma -2\left(1-\gamma \right)\theta \varphi v}{2\left[1-\left(1-\gamma \right)\theta \varphi v\right]\left(1-\theta \varphi v\right)},B=\frac{2}{\varphi -\theta v},C=\frac{2-\gamma }{2\left[1-\left(1-\gamma \right)\varphi v\right]},D=\frac{1-\gamma }{1-\left(1-\gamma \right)\varphi v},and\;H=AB+\left(v-\varphi \right)AD+BD$

**Figure 1:**
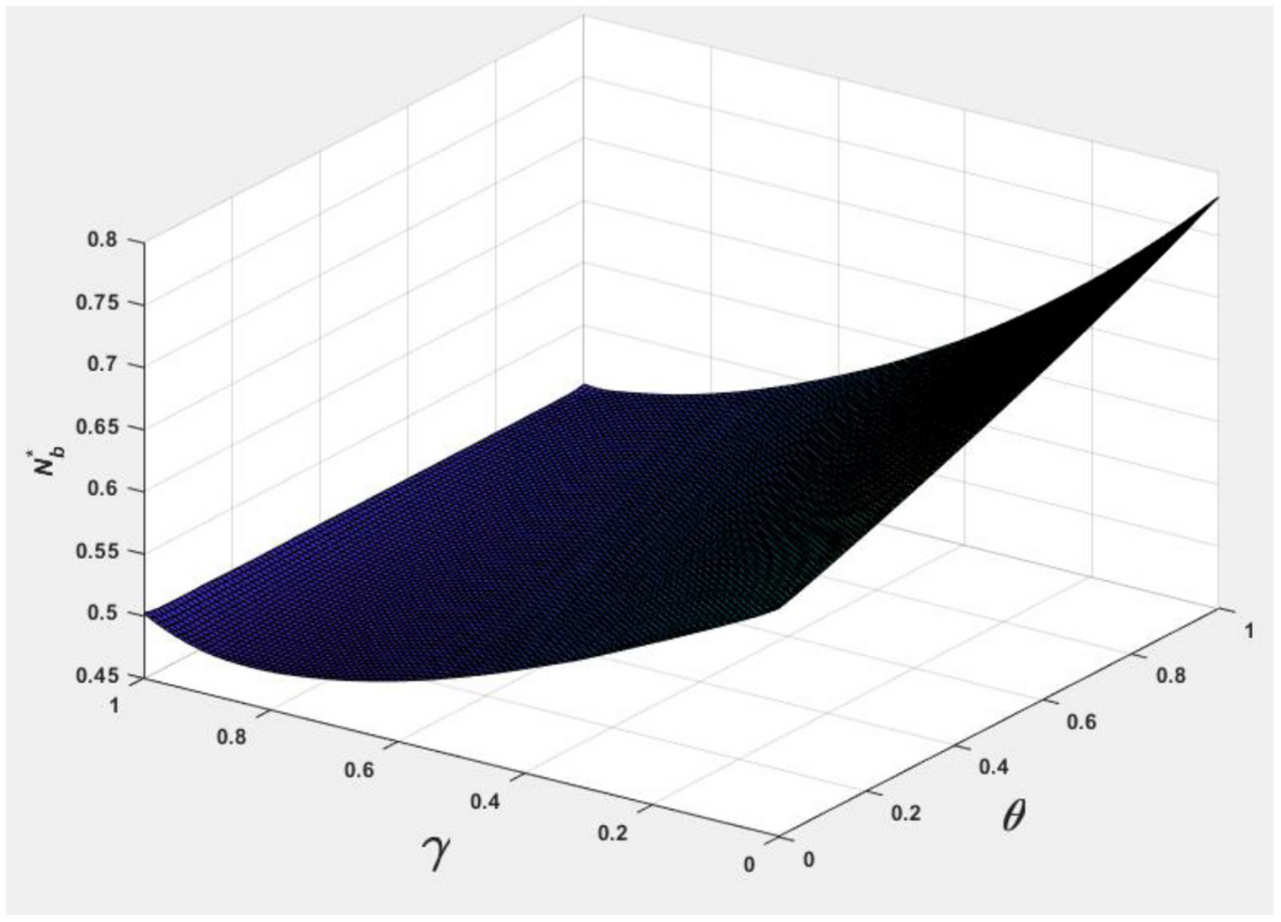
Effect of θ, γ on $N_{b}^{*}$.

**Figure 2:**
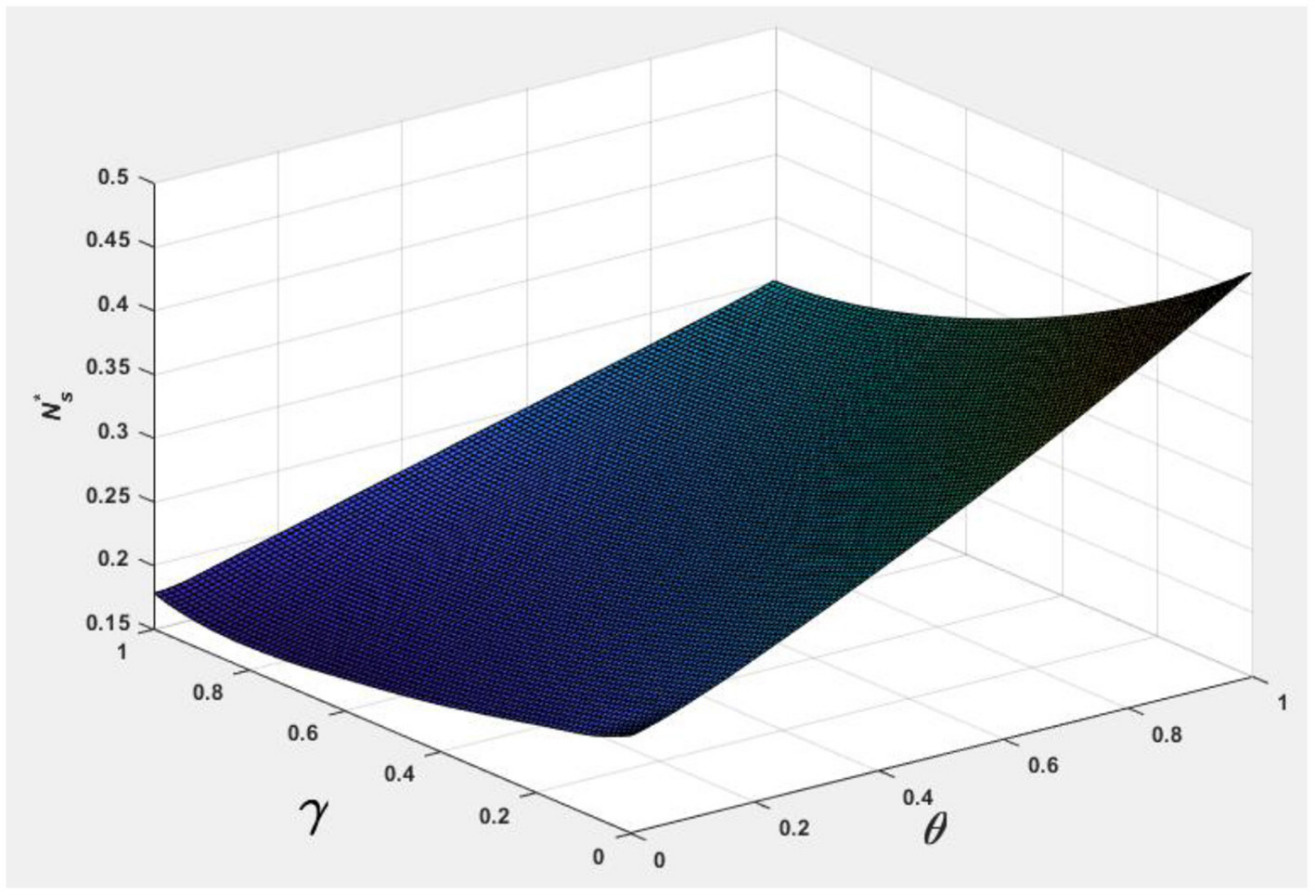
Effect of θ, γ on $N_{s}^{*}$.

**Figure 3:**
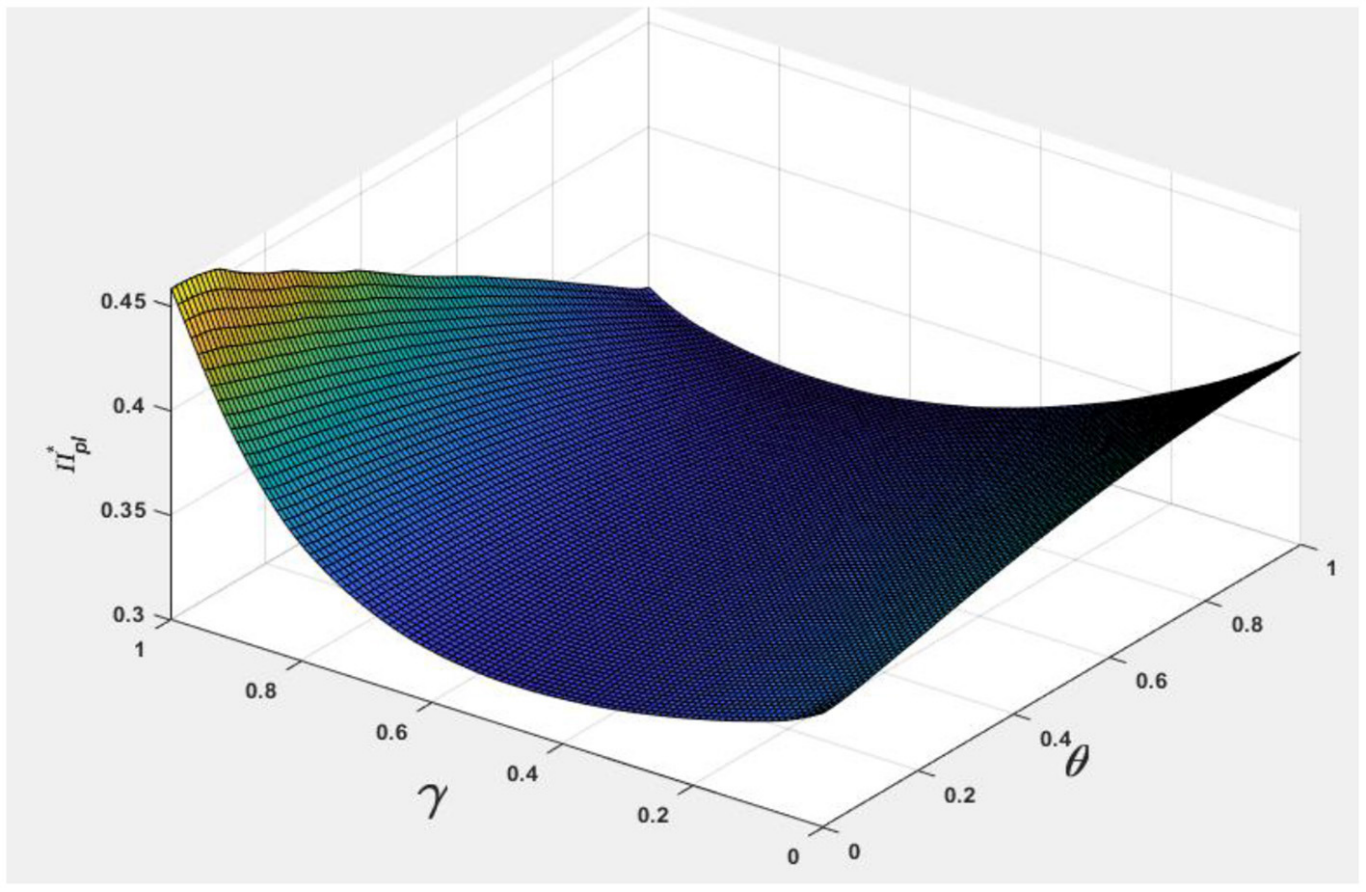
Effect of θ, γ on $\Pi_{pl}^{*}$.

**Figure 4:**
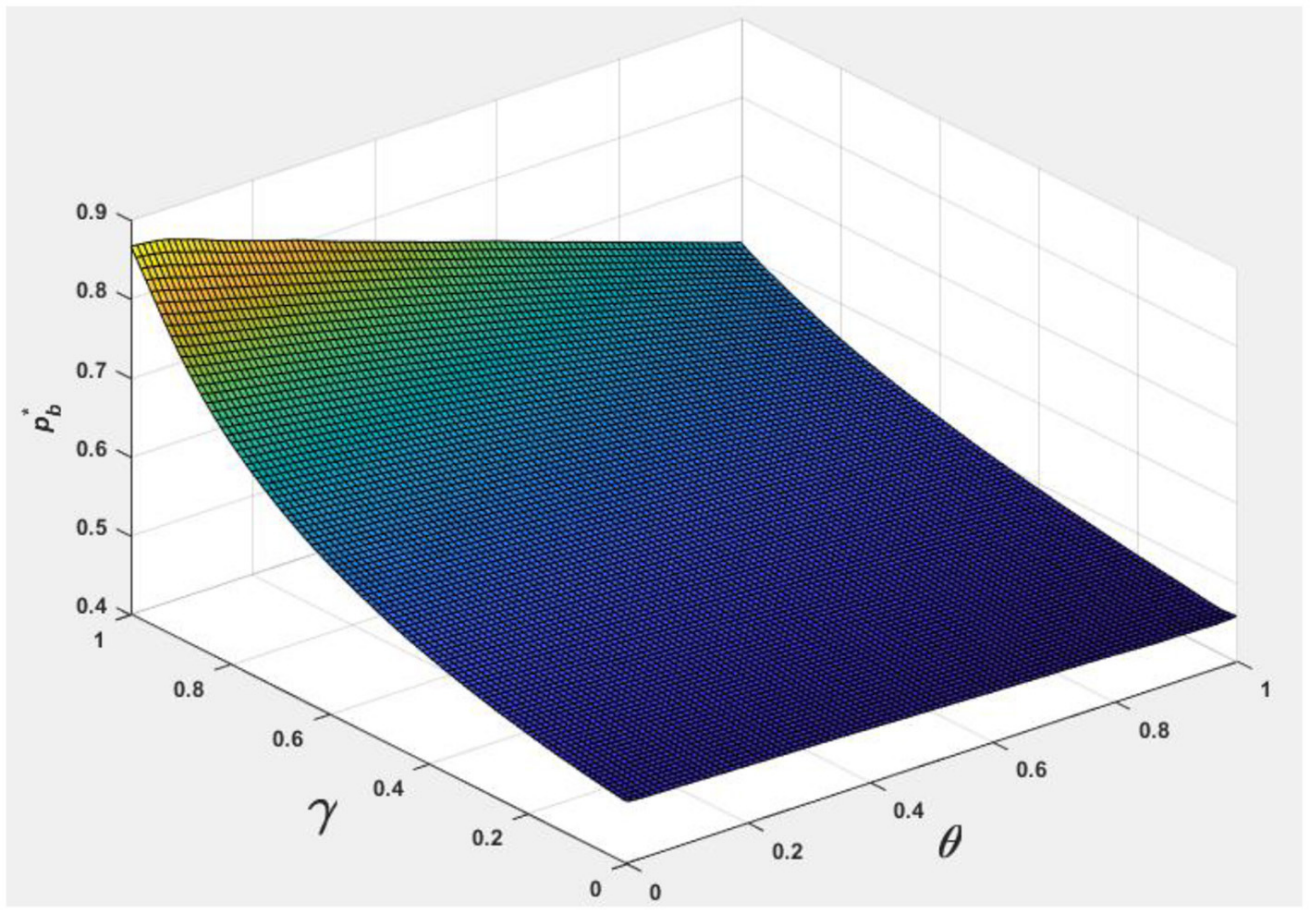
Effect of θ, γ on $p_{b}^{*}$.

**Figure 5:**
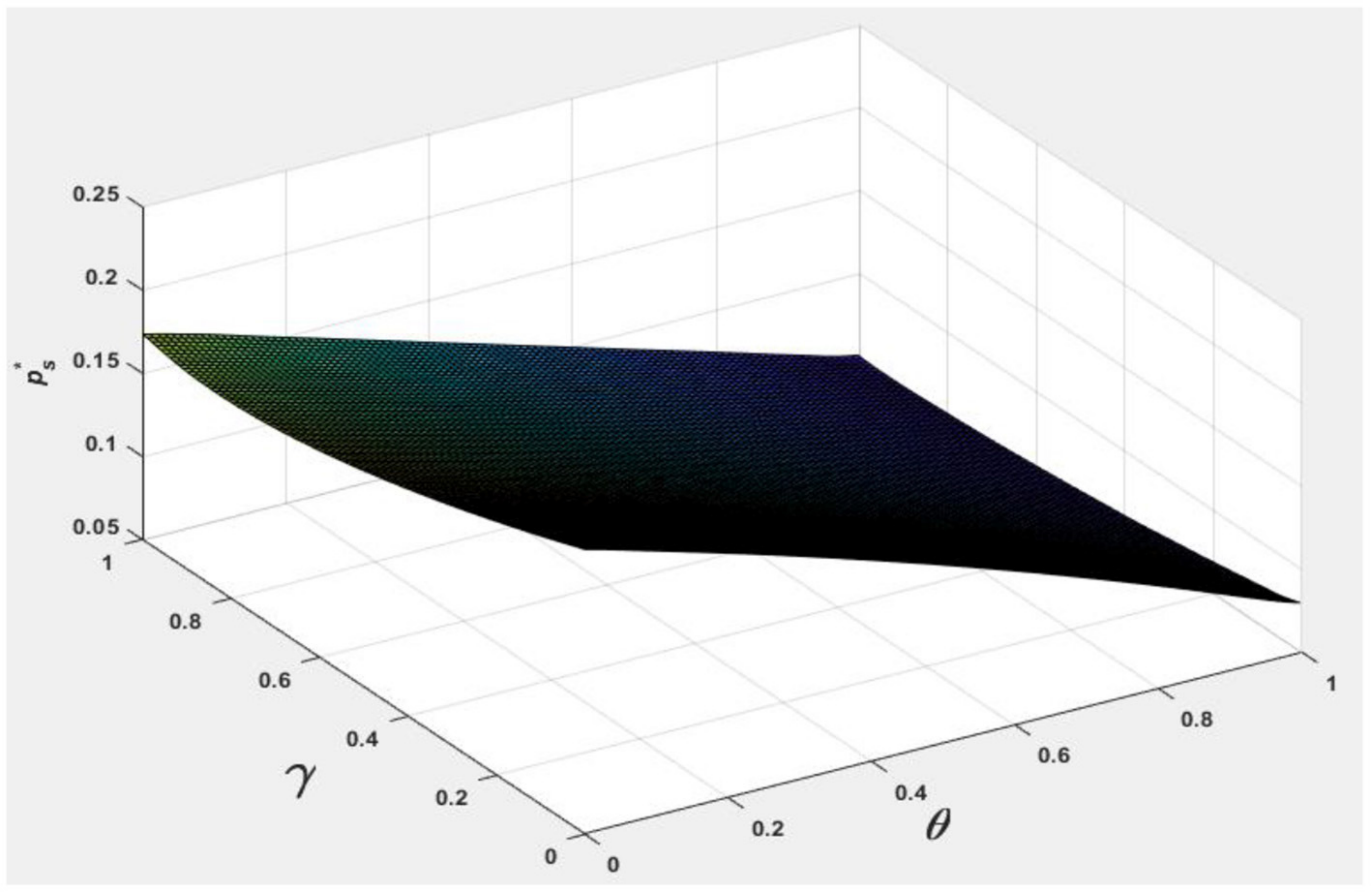
Effect of θ, γ on $p_{s}^{*}$.

To facilitate analyzing the effects of consumer information level θ and the market competition level γ on $p_{b}^{*}$, $p_{s}^{*}$, $N_{b}^{*}$, $N_{s}^{*}$, and $\Pi_{pl}^{*}$, we let *v* = 0.5 and φ = 0.7, and the following Figures 1–5 are obtained by referring to the assignment of Hagiu and Hałaburda (2014).

From Figures 1, 2, it can be seen that to occupy a larger market share, eBay, Taobao, JD, Guazi, and other online shopping platforms provide better differentiated return services to improve consumer information levels, such as Tmal (with a 30-day warranty service) and Nike (with a 30-day no-reason return service) (Choi, 2013). JD has launched 1 and 3-year warranty services. Guazi provides a 30-day comprehensive warranty, a 7-day no-reason return, a 7-day no-reason exchange, a 259 safety inspection, and other services to protect consumer rights.

From Figure 3, to obtain greater profits, if the market competition level is lower, platforms have more incentive to improve consumer information level. In contrast, when market competition level is higher, platforms have less incentive to improve the level of information services. Therefore, the market generally adopts a 7-day no-reason return service for most competitive goods in online shopping platforms.

From Figures 4, 5, it can be seen that to improve consumer information level, the more competitive the platform is, the lower the membership fees charged to consumers and merchants. We see Taobao and JD, for example, give consumers red packet allowances to improve the size of transactions s. They also give different forms of subsidies such as technical service fees and free store decorations to merchants.

## Discussion

The Armstrong (2006) model introduces cross-network externalities, and explains why online shopping platforms adopt skewed pricing strategies to subsidize consumers. Hagiu and Hałaburda (2014) introduce expectation factors and changes in cross-network externalities under different expectation formation mechanisms, leading to different responses in platform pricing, which ultimately lead to differences in platform profits. In the monopoly platform, profits of responsive expectations are higher than those of passive expectations; in the competitive platform, platform profits of passive expectations are higher than those of responsive expectations. Based on Armstrong (2006) and Hagiu and Hałaburda (2014), our research develops an imperfect information pricing model considering the level of consumer information to explain why online shopping platforms adopt measures to reduce the level of bilateral information asymmetries. This section further examines the effect of the platform increasing consumer information level (e.g., adopting return measures) on platform pricing, the number of bilateral users, and platform profits.

Guazi, eBay, Taobao, JD, and other online trading platforms provide a basic level of information to consumers. The platforms adopt return measures to release buyers and sellers from the contract to protect consumers' rights and maximize profits. This approach motivates merchants to disclose more private information about their goods, reduces the information asymmetry of bilateral users, and acts as a disincentive for dishonest merchants to trade. When the platform adopts return measures, the consumer information level function is shown as Equation (33):


(33) $$\begin{array}{l} \theta =k\omega \;(t)\;+\;\theta_{0} \end{array}$$

Here, *k* > 0, *k* denotes the conversion coefficient between platform return service level and consumer information level; ω (*t*) (ω (*t*) ∈ [0, 1]) denotes platform return service level, and whenω (*t*) = 0, the online shopping platform does not adopt return measures. This study assumes that the longer the return time provided by the platform, the higher the platform return service level when consumers return goods purchased that do not meet their expectations, i.e., $\frac{\partial \omega \left(t\right)}{\partial t}\geq \;0$.

To facilitate transactions, merchants in online shopping platforms use pictures, text, videos, live streaming, and other forms to convey product information to consumers on the product display page. However, consumers have different levels of information about each type of goods before a purchase. Nelson (1970) divided goods into two categories: search goods and experience goods. On the premise that consumer information level and conversion coefficient remain unchanged, consumers have more information about search goods (such as clothes, pants, and shoes) than experience goods (such as cars and food) before shopping, i.e., θ_0(*Search goods*)_ > θ_0(*Experience goods*)_. When consumers return goods that do not meet their expectations, the platform sets the return time for search goods shorter than that for experience goods. For example, eBay has different return times for fashion, health and beauty, home and garden, media and other goods, and return times are at least 30 or 60 days.

### Analyzing the Role of the Monopoly Platform When Adopting Return Measures

From Equation (14), when no return measures are adopted, the monopoly platform pricing for consumers and merchants is given, as shown in Equation (34):


(34) $$\begin{array}{l} p_{b}^{1}=\frac{2-\varphi \;(\varphi +\theta_{0}v)\;}{4-\;(\varphi +v)\;\;(\varphi +\theta_{0}v)\;},p_{s}^{1}=\frac{\varphi -\theta_{0}v}{4-\;(\varphi +v)\;\;(\varphi +\theta_{0}v\;)\;} \end{array}$$

From Equation (15), when no return measures are adopted, the number of consumers and merchants is given in the monopoly platform, as shown in Equation (35):


(35) $$\begin{array}{l} N_{b}^{1}=\frac{2}{4-\;(\varphi +v)\;\;(\varphi +\theta_{0}v)\;},N_{s}^{1}=\frac{\varphi +\theta_{0}v}{4-\;(\varphi +v)\;\;(\varphi +\theta_{0}v\;)\;} \end{array}$$

From Equation (16), we can obtain monopoly platform profit when no return measures are adopted, as shown in Equation (36):


(36) $$\begin{array}{l} \Pi_{pl}^{1}=\frac{4-{\;(\varphi +\theta_{0}v)\;}^{2}}{{\left[4-\;(\varphi +v)\;\;(\varphi +\theta_{0}v)\;\;\right]}^{2}} \end{array}$$

Substituting Equation (33) into Equation (14), we obtain the pricing for consumers $p_{b}^{2}$ and pricing for merchants $p_{s}^{2}$ when the monopoly online shopping platform adopts return measures and then compare them with the pricing for consumers $p_{b}^{1}$ and pricing for merchants $p_{s}^{1}$ when no return measures are adopted, as shown in Equation (37):


(37) $$\begin{array}{l} \frac{p_{b}^{2}}{p_{b}^{1}}=\frac{8-4\varphi \;(\varphi +\theta_{0}v)\;-2\;(\varphi +v)\;\;(\varphi +\theta_{0}v)\;+\varphi \;(\varphi +v)\;\;(\varphi +\theta_{0}v)\;\;(\varphi +\theta_{0}v+k\omega v)\;-4\varphi k\omega v}{8-4\varphi \;(\varphi +\theta_{0}v)\;-2\;(\varphi +v)\;\;(\varphi +\theta_{0}v)\;+\varphi \;(\varphi +v)\;\;(\varphi +\theta_{0}v)\;\;(\varphi +\theta_{0}v+k\omega v)\;-2\;(\varphi +v)\;\;k\omega v}\;\\ \frac{p_{s}^{2}}{p_{s}^{1}}=\frac{4\;(\varphi -\theta_{0}v)\;-\varphi \;(\varphi +v)\;\;(\varphi +\theta_{0}v)\;+v\;(\varphi +v)\;\theta_{0}\;(\varphi +\theta_{0}v+k\omega v)\;+\left[\varphi \;(\varphi +v)\;-4\right]k\omega v}{4\;(\varphi -\theta_{0}v)\;-\varphi \;(\varphi +v)\;\;(\varphi +\theta_{0}v)\;+v\;(\varphi +v)\;\theta_{0}\;(\varphi +\theta_{0}v+k\omega v)\;-\varphi \;(\varphi +v)\;\;k\omega v}<1 \end{array}$$

From Equation (37), when merchant-to-consumer network externalities are greater than consumer-to-merchant network externalities, $\frac{p_{b}^{2}}{p_{b}^{1}}>1$ is given. When the monopoly online shopping platform adopts return measures, it raises charges to consumers (e.g., reduces subsidies). Conversely, the monopoly online shopping platform reduces charges to consumers (e.g., the platform will subsidize consumers on top of original charges). When the monopoly online shopping platform adopts return measures (increasing consumer information level), the platform will reduce charges to merchants.

Substituting Equation (33) into Equation (15), we obtain the number of consumers $N_{b}^{2}$ and the number of merchants$N_{s}^{2}\;$when the monopoly online shopping platform adopts return measures and then compare them with the number of consumers $N_{b}^{1}$ and the number of merchants $N_{s}^{1}$ when return measures are not adopted, as shown in Equation (38):


(38) $$\begin{array}{l} \frac{N_{b}^{2\;}}{N_{b}^{1}}=\frac{4-\;(\varphi +v)\;\;(\varphi +\theta_{0}v)\;}{4-\;(\varphi +v)\;\;(\varphi +\theta_{0}v+k\omega v)\;}>\;1,\\ \frac{N_{s}^{2}}{N_{s}^{1}}=\frac{\begin{array}{l} 4\;(\varphi +\theta_{0}v)\;-\varphi \;(\varphi +v)\;\;(\varphi +\theta_{0}v)\;-v\;(\varphi +v)\;\theta_{0}\\ \;(\varphi +\theta_{0}v+k\omega v)\;-\varphi \;(\varphi +v)\;\;k\omega v+4k\omega v \end{array}}{\begin{array}{l} 4\;(\varphi +\theta_{0}v)\;-\varphi \;(\varphi +v)\;\;(\varphi +\theta_{0}v)\;-v\;(\varphi +v)\;\theta_{0}\\ \;(\varphi +\theta_{0}v+k\omega v)\;-\varphi \;(\varphi +v)\;\;k\omega v \end{array}}>\;1 \end{array}$$

From Equation (38), when the monopoly online shopping platform adopts return measures, it attracts more consumers to enter the platform to trade. With cross-network externalities, it attracts more merchants to trade on the platform.

Substituting Equation (33) into Equation (16), we obtain the profit $\Pi_{pl}^{2}$ when the monopoly online shopping platform adopts return measures and then compare it with the profit $\Pi_{pl}^{1}$ when the return measure is not adopted, as shown in Equation (39):


(39) $$\begin{array}{l} \frac{\Pi_{pl}^{1}}{\Pi_{pl}^{2}}=\frac{4-{\;(\varphi +\theta_{0}v)\;}^{2}}{4-{\;(\varphi +\theta_{0}v+k\omega v)\;}^{2}}*{\left[\frac{4-\;(\varphi +v)\;\;(\varphi +\theta_{0}v+k\omega v)\;}{4-\;(\varphi +v)\;\;(\varphi +\theta_{0}v)\;}\right]}^{2}\\ \;<\;1 \end{array}$$

From Equation (39), when the monopoly online shopping platform adopts return measures, it can promote the growth of platform profits.

### Analyzing the Role of the Competitive Platform When Adopting Return Measures

From Equation (24), when no return measures are adopted, the competitive platform pricing for consumers and merchants is given, as shown in Equation (40):


(40) $$\begin{array}{l} p_{b}^{3}=\frac{4-3\theta_{0}\varphi v-\varphi^{2}}{4},p_{s}^{3}=\frac{\varphi -\;\theta_{0}v}{4} \end{array}$$

From Equation (25), when no return measures are adopted, the number of consumers and merchants is given in the competitive platform, as shown in Equation (41):


(41) $$\begin{array}{l} N_{b}^{3}=\frac{1}{2},N_{s}^{3}=\frac{\varphi +\;\theta_{0}v}{4}\;(41)\;. \end{array}$$

From Equation (26), we can obtain competitive platform profit when no return measures are adopted, as shown in Equation (42):


(42) $$\begin{array}{l} \Pi_{pl}^{3}=\frac{8-\varphi^{2}-6\theta_{0}\varphi v-\;{\theta_{0}}^{2}v^{2}}{16} \end{array}$$

Substituting Equation (33) into Equation (24), we obtain the pricing for consumers $p_{b}^{4}$ and pricing for merchants $p_{s}^{4\;}$when the competitive online shopping platform adopts return measures, and then we subtract, respectively, the pricing for consumers $p_{b}^{3}$ and pricing for merchants $p_{s}^{3}$ when the competitive online shopping platforms do not adopt return measures, as shown in Equation (43):


(43) $$\begin{array}{l} p_{b}^{4}-p_{b}^{3}=\frac{-3k\omega \varphi v}{4}<0,p_{s}^{4}-p_{s}^{3}=\frac{-k\omega v}{4}<\;0 \end{array}$$

From Equation (43), when the competitive online shopping platform adopts return measures, the platform reduces consumer and merchant charges.

Substituting Equation (33) into Equation (25), we obtain the number of consumers $N_{b}^{4}$ and the number of merchants $N_{s}^{4}$ when the competitive online shopping platform adopts return measures, and then subtract, respectively, the number of consumers $N_{b}^{3}$ and the number of merchants $N_{s}^{3}$ when the competitive online shopping platform does not adopt return measures, as shown in Equation (44):


(44) $$\begin{array}{l} N_{b}^{4}-N_{b}^{3}=0,N_{s}^{4}-N_{s}^{3}=\frac{k\omega v}{4}>\;0. \end{array}$$

From Equation (44), the competitive online shopping platform maintains consumer size and attracts more merchants to join platform transactions when it adopts return measures.

Substituting Equation (33) into Equation (26), we obtain the profit $\Pi_{pl}^{4}$ when the competitive online shopping platform adopts return measures, and then subtract the profit $\Pi_{pl}^{3}$ when the competitive online shopping platform does not adopt return measures, as shown in Equation (45):


(45) $$\begin{array}{l} \Pi_{pl}^{4}-\Pi_{pl}^{3}=\frac{-6k\omega \varphi v-{\;(k\omega )\;}^{2}v^{2}-2k\omega \theta_{0}v^{2}}{16}<\;0 \end{array}$$

From Equation (45), when the competitive online shopping platform adopts return measures, it sacrifices part of its profit to subsidize merchants and consumers, maintaining the number of consumers and expanding the number of merchants.

## Conclusion

Online shopping platforms bring convenience to consumers, but there are still dishonest trading problems, such as mismatches between transaction price and expected quality of goods purchased. For long-term development, online shopping platforms, such as Taobao and JD, often adopt 7-day no-reason return services; likewise, 30-day no-reason return measures are used by Nike (Choi, 2013). Also, cash-on-delivery, word-of-mouth reviews, merchant ratings, and other measures reduce bilateral user information asymmetry level and govern dishonest transactions. In this study, based on previous research (Armstrong, 2006; Hagiu and Hałaburda, 2014), we constructed a pricing model for online shopping platforms, which introduces consumer information level, and we analyze pricing strategies of monopoly and competitive online shopping platforms. Furthermore, we examined the role of online shopping platforms when adopting return measures. To assess the robustness of the findings, we study the pricing strategies of online shopping platforms under imperfect competition conditions in the market. We obtain the following conclusions under the consideration of network effects.

(1) In the monopoly online shopping platform, the lower the bilateral user information asymmetry level, merchants and consumers that are attracted to trade, and platform profits are higher. Therefore, monopoly platforms have an incentive to improve consumer information level when they pursue profit maximization.

When the monopoly online shopping platform increases consumer information level (e.g., adopting return measures), the platform can reduce charges to merchants. The platform subsidizes merchants on original charges, e.g., by reducing merchant registration fees, reducing technical service fees, providing free store decorations, or similar measures. Thus, the platform can attract more merchants and consumers to trade and can realize platform profit growth.

(2) The higher the consumer information level in the competitive online shopping platform, the lower the fees charged to merchants and consumers. The platform sacrifices some of its profits to attract more merchants to join and to maintain a number of consumers. Therefore, relative to the monopoly platform, the competitive platform has relatively little incentive to increase consumer information level while pursuing profit maximization.

Since competitive online shopping platforms have little incentive to improve consumer information level, market regulators should adopt a 14-day return time (or longer) to protect consumer rights. When a competitive online shopping platform increases consumer information level (e.g., by adopting return measures), it can expand the number of merchants and maintain the number of consumers by reducing charges for bilateral users. For consumers, the platform can issue red packets, discount coupons, etc.; for merchants, the platform can provide free value-added services and other similar benefits.

(3) Since consumers have different levels of information about various goods, online shopping platforms adopt different high-quality return services to reduce bilateral user information asymmetry level, and the return time of experience goods is greater than that of search goods.

The study also has some shortcomings. For example, merchants assume complete information about consumers, and consumers do not adjust their decisions based on available information. We further study platform pricing strategies with bilateral information uncertainty between merchants and consumers.

## Data Availability Statement

The original contributions presented in the study are included in the article/supplementary material, further inquiries can be directed to the corresponding author/s.

## Author Contributions

HC: conceptualization, methodology, visualization, investigation, supervision, and writing (original draft). WX: software, data curation, validation, and writing (review and editing). PX: conceptualization, project administrations, supervision, validation, and writing (review and editing). All authors contributed to the article and approved the submitted version.

## Funding

This research was supported by the National Social Science Foundation of China (No. 21BJY072), the National Natural Science Foundation of China (No. 71771128), and the Zhejiang University of Finance and Economics Dongfang College Project (No. 2021dfy022).

## Conflict of Interest

The authors declare that the research was conducted in the absence of any commercial or financial relationships that could be construed as a potential conflict of interest.

## Publisher's Note

All claims expressed in this article are solely those of the authors and do not necessarily represent those of their affiliated organizations, or those of the publisher, the editors and the reviewers. Any product that may be evaluated in this article, or claim that may be made by its manufacturer, is not guaranteed or endorsed by the publisher.
